# Hereditary Pancreatic Cancer: Advances in Genetic Testing, Early Detection Strategies, and Personalized Management

**DOI:** 10.3390/jcm14020367

**Published:** 2025-01-09

**Authors:** Carmen Blanco Abad, Paula Gomila Pons, Sara Campos Ramírez, María Álvarez Alejandro, María Irene Torres Ramón, María Dolores Miramar Gallart, Silvia Izquierdo Álvarez, Eduardo Polo Marques, Roberto Pazo Cid

**Affiliations:** 1 Medical Oncology Department, Hospital Universitario Miguel Servet, 50012 Zaragoza, Spain; 2 Aragon Institute of Health Sciences (IIS-A), 50012 Zaragoza, Spain; 3 Medical Oncology Department, Hospital Clinico Universitario Lozano Blesa, 50009 Zaragoza, Spain; 4 Genetics Unit, Biochemistry Department, Hospital Universitario Miguel Servet, 50012 Zaragoza, Spain; 5 Department of Medicine, Psychiatry and Dermatology, Faculty of Medicine, Zaragoza University, 50009 Zaragoza, Spain

**Keywords:** pancreatic ductal adenocarcinoma, hereditary cancer, genetic testing, biomarkers, early detection, miRNA, hyperpolarized 13C-MRS, multidisciplinary management

## Abstract

**Background**: Pancreatic ductal adenocarcinoma (PDAC) is a highly lethal malignancy with a five-year survival rate of approximately 13% for advanced stages. While the majority of PDAC cases are sporadic, a significant subset is attributable to hereditary and familial predispositions, accounting for approximately 25% of cases. This article synthesizes recent advancements in the understanding, detection, and management of hereditary pancreatic cancer (PC). **Results**: Our review highlights the critical role of genetic testing (GT) in identifying high-risk individuals (HRIs), with germline pathogenic variants (PVs) found in up to 20% of hereditary PDAC cases. Since the implementation of next-generation sequencing (NGS) panels in 2014, detection capabilities have been significantly enhanced. HRIs can be included in screening programs that facilitate the early detection of PDAC. Early detection strategies, including the use of microribonucleic acid (miRNAs) signatures and novel imaging techniques like hyperpolarized 13C-magnetic resonance spectroscopy (MRS) have shown promising results. The identification of germline pathogenic variants (PVs) or mutations in homologous recombination (HR) genes plays a predictive role in the response to various treatments, prolonging patient survival. **Discussion**: Universal germline testing for PDAC, as recommended by the National Comprehensive Cancer Network (NCCN), is now a standard practice, facilitating the identification of at-risk individuals and enabling targeted surveillance and intervention. Multidisciplinary management, integrating genetic counseling, imaging, and gastrointestinal services, is essential for optimizing outcomes. **Conclusions**: Advances in genetic testing and biomarker research are transforming the landscape of hereditary PC management. Early detection and personalized treatment strategies are pivotal in improving survival rates. Ongoing multi-institutional research efforts are crucial for validating biomarkers and developing preventive measures, ultimately aiming to reduce the burden of this aggressive cancer.

## 1. Introduction

PDAC is a significant health burden. Its incidence is projected to increase, making it the second leading cause of cancer-related deaths by 2030 [1]. Early detection is crucial, as surgery represents the only curative treatment option, while the prognosis of patients diagnosed at stage IV remains poor [1,2]. However, most PDAC tumors are diagnosed at an advanced or locally advanced stage [2].

Given that typical risk factors, such as age or personal and familial history of cancer, are not reliable predictors of hereditary PC, GT has been recommended for all patients diagnosed with PDAC since 2019 [3]. Despite these recommendations, GT remains underutilized [1]. As a result, various strategies are being explored to increase the number of patients undergoing GT.

Identifying families carrying germline mutations in genes predisposing to PDAC and families meeting the criteria for familial pancreatic cancer (FPC) enables the identification of HRIs predisposed to developing PDAC. FPC criteria are defined as at least two first-degree relatives affected by PDAC without an identifiable hereditary syndrome [4,5,6]. Identifying these HRIs provides an opportunity to enroll them in screening programs. However, the selection of HRIs who may benefit from screening programs and surveillance strategies remains a challenge.

This review aims to summarize the evidence on mutations associated with hereditary PC and FPC, the current recommendations for GT in PDAC, the available evidence on PC screening in hereditary PC and methods to enhance its efficacy, as well as the prognostic and predictive implications of homologous recombination deficiency (HRD) mutations. Herein, for the purpose of this review, we will focus specifically on PDAC, which accounts for 95% of PC [6]. Other rarer pancreatic malignancies, such as squamous carcinoma, neuroendocrine tumors, and colloid carcinoma, are beyond the scope of this review.

## 2. Results

### 2.1. Risk Factors for PDAC

Risk factors can be divided into modifiable and non-modifiable risk factors.

#### 2.1.1. Non-Modifiable Risk Factors

- Non-hereditary risk factors

PDAC incidence increases significantly with age and is frequently diagnosed in patients between 60 and 80 years of age. This age-related increase may be explained by mitochondrial electron transport chain dysfunction, which leads to the accumulation of oxygen radicals that damage cellular components [7].

The influence of sex on PDAC risk is also notable. The incidence of PDAC is higher in men, potentially due to a greater prevalence of exposure to modifiable risk factors [7].

Emerging evidence suggests that height and blood group may also influence PDAC risk; however, the exact mechanisms remain unclear [7].

- Genetic Mutations Linked to Hereditary PDAC

In patients diagnosed with “sporadic” PDAC, the incidence of germline mutations ranges from 3.9% to 19.8%, according to various studies [8,9,10,11,12,13]. Notably, higher incidences of germline PVs have been observed in certain ethnic groups. For example, Ashkenazi Jewish individuals show a *BRCA1/2* mutation prevalence of up to 15%, while African Americans exhibit a slightly lower, yet still significant, incidence of *BRCA* mutations, ranging from 7% to 13% [8].

Younger patients appear to harbor more germline mutations. Among those diagnosed before the age of 60, germline PVs are found in 21.2% of cases [9], emphasizing the importance of GT in this population, regardless of family history.

Genes identified in hereditary PDAC play a crucial role in the development of the disease. These genes may serve as prognostic factors of disease progression and help identify patients who are candidates for specific oncological treatments [10].

A wide variety of genes have been implicated in hereditary PDAC. In this section, we will review the most common genetic mutations associated with hereditary PDAC.

- *BRCA1/2*

These tumor suppressor genes are associated with a variety of carcinomas, including breast, ovarian, prostate, and PDAC [11]. The incidence of PVs in these genes in the germline ranges from 5% to 9% [12]. Carriers of these mutations have an increased relative risk of developing PDAC, with a relative risk of 2.26 for *BRCA1* mutation carriers and 3.5–10 for *BRCA2* mutation carriers [10].

Patients with *BRCA1/2* PVs differ from patients with sporadic forms of PDAC [13]. The median age of diagnosis is 62.9 years, which is a decade younger than the median age of diagnosis in the general population, as reported in the Surveillance, Epidemiology, and End Results database [14]. There is conflicting evidence regarding whether the presence of germline *BRCA1/2* mutations has an impact on prognosis [13].

A cohort study that included 71 *BRCA*-positive PDAC patients found that *BRCA*-mutant patients had a better prognosis than the general PDAC population [14]. The authors suggested that this improvement may be related to the younger age of patients with PDAC carrying a germline mutation [14]. Therefore, the detection of PDAC at earlier stages is crucial, and new treatment strategies targeting this specific population need to be considered, as discussed further in this article [15,16].

b. Lynch Syndrome-associated Genes

Lynch Syndrome (LS) is an autosomal dominant disease produced by germline mutations in mismatch repair (MMR) genes (such as *MLH1*, *PMS2*, *MSH2,* and *MSH6*) or germline mutations in epithelial cell adhesion molecules (*EPCAM*) [17]. All these alterations cause microsatellite instability (MSI) due to a reduced capacity to produce MMR proteins and an inability of cells to correct nucleotide mismatches during deoxyribonucleic acid (DNA) replication [18].

Clinically, PDAC occurs in patients with LS type II. The incidence of PDAC in LS patients ranges from 1.3% to 4% [18]. The age of presentation does not significantly differ from sporadic PDAC, but it does vary from other tumors associated with LS. The reason for this finding is not well understood [18,19,20]. The majority of LS patients have a medical history of multiple cancers [19].

In general, MSI is a better prognostic factor for PDAC, potentially due to the stronger anti-tumoral response of the immune system in these patients [20]. Increased lymphocyte infiltration and PD-1 and PD-L1 expression are observed in PDAC tumors with MSI compared to those without MSI [21]. The implications of this finding for treatment selection will be discussed in more detail later in this article. Patients with MSI-PDAC tend to be less responsive to fluorouracil and gemcitabine but show greater responsiveness to FOLFIRINOX [20,21].

c. Other germline PVs: *CDKN2A*, *APC*, *STK11*, *TP53*, *ATM*, *PALB2*, *PRSS1*, and *CFTR*

**Figure 1:**
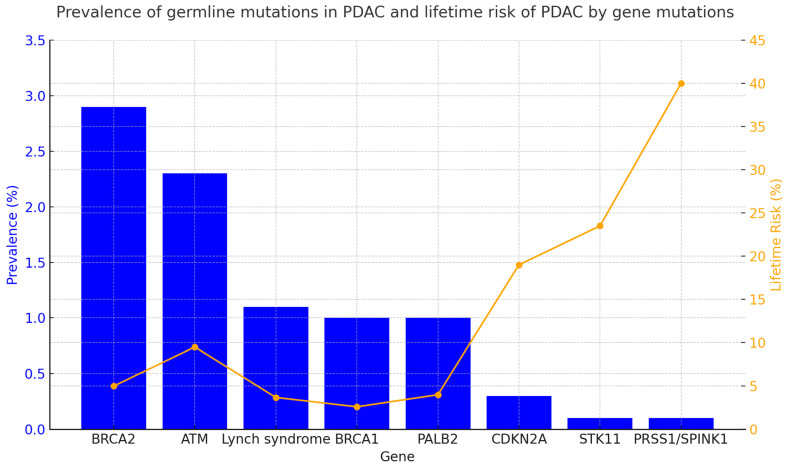
Prevalence and lifetime risk of pancreatic cancer by gene [4,28,29,30,31]. The figure summarizes the prevalence (%) of germline mutations in patients diagnosed with PDAC and the lifetime risk (%) of developing PDAC in individuals carrying germline mutations in specific predisposing genes. Data were collected from various studies referenced in the figure. When multiple data points were reported, the median of the published ranges was used.

Other less frequent germline mutations (<1–5%) are associated with an increased risk of PDAC, including *CDKN2A*, *APC*, *STK11*, *TP53*, *ATM*, *PALB2*, *PRSS1*, and *CFTR*. Each of these mutations causes a clinical hereditary syndrome that is associated with other clinical characteristics. A summary of these characteristics is provided in Table 1. Figure 1 presents the incidence of germline mutations in PDAC patients and the lifetime risk (%) of developing PDAC in individuals carrying these genetic mutations.

**Table 1:** Less frequent germline mutations associated with familial PDAC.

PV	Clinical Syndrome	Pattern of Inheritance	Risk of PDAC	Other Characteristics	References
*CDKN2A*	Familial atypical multiple melanoma syndrome (FAMMM)	Autosomal dominant	Cumulative risk of 17% and relative risk of 13–39	Increased risk of melanoma in 70%	[10,22,23,24]
*APC*	Adenomatous polyposis syndrome	Autosomal dominant	Relative risk of 4.46; cumulative risk of 2%	Hundreds to thousands of adenomatous polyps, increased risk of colorectal cancer in 100% of carriers.	[10,24,25]
*STK11*	Peutz–Jeghers syndrome	Autosomal dominant	Cumulative risk of 11–36%, relative risk of 76.3	Mucocutaneous pigment macules and hamartomatous GI polyps, mean age of onset of PDAC is 40.8 years	[10,22]
*TP53*	Li–Fraumeni syndrome	Autosomal dominant	Cumulative risk of 1.1–9.5%, relative risk 2.41–6.5.	Ataxia, telangiectasias, immunosuppression, and an increased risk for leukemia and lymphoma, breast, ovarian, prostate, and other cancers	[10,22,23,24,26]
*PALB2*		Autosomal recessive	Cumulative risk of 2–3%, relative risk of 2.37	Monoallelic mutations predispose to breast, ovarian, and pancreatic familial cancers; increased sensitivity to platinum agents.	[10,22,23]
*ATM*	Ataxia–telangiectasia	Autosomal recessive	Relative risk of 2.41	Increased risk of breast, ovarian, and prostate cancer; reduced sensitivity to gemcitabine	[10,22,24,27,28]
*PRSS1/SPINK1/CFTR*	Hereditary pancreatitis	Autosomal dominant	PRSS1 and SPINK1: lifetime risk ranged between 18.8% and 53.3% CFTR: relative risk of 2.9–4.5	Linked to acute and chronic pancreatitis	[10,22,29,30]

Less frequent gene PVs and their different characteristics, including risk of PDAC, pattern of inheritance, and other clinical characteristics, are presented. GI: gastro-intestinal.

- Familial pancreatic cancer

FPC is defined as a familial cluster of PDAC with at least two first-degree relatives without a known hereditary syndrome [10]. Among patients who meet the criteria for FPC, germline PVs are found in only 10–20% of cases [32], suggesting that there are still undiscovered genes related to hereditary predisposition to PDAC.

Individuals who meet the criteria of FPC have a 9.0-fold higher (95% CI, 4.5–16.1) risk of developing PDAC [33]. The risk is especially high in those with three or more first-degree relatives affected, with a 32.0-fold increase in risk (95% CI, 10.4–74.7) [32]. Smoking significantly increases the risk of PDAC in families with FPC [33]. In addition, having a relative diagnosed with PDAC at an early age (<50 years) further increases the risk of developing PDAC [34]. The cumulative risk of developing PDAC by the age of 80 years is 15.7% in individuals with one or two relatives diagnosed at age 40 and 38.5% in those with three relatives diagnosed at age 40 [34].

#### 2.1.2. Modifiable Risk Factors

Tobacco use doubles the risk of PDAC compared to nonusers in the general population [6]. Smoking is associated with increased inflammatory responses in the pancreas, driven by carcinogenic compounds such as N-nitrosamines and aromatic hydrocarbons. These carcinogens can mutate both proto-oncogenes and tumor suppressor genes [7].

Excessive alcohol intake (≥3 drinks/day) modestly increases PDAC risk, with RRs ranging from 1.16 to 1.19, driven by alcohol-induced inflammation and chronic pancreatitis [6]. Obesity also elevates PDAC risk, with an RR of 1.72 for individuals with a body mass index (BMI) ≥ 30 kg/m^2^ compared to those with a BMI < 23 kg/m^2^ [6]. Other dietary factors, such as high intake of red or processed meats, further contribute to PDAC risk, whereas regular physical activity and a diet rich in fruits and vegetables are protective [4].

PDAC risk factors significantly alter the baseline risk in individuals with hereditary predispositions. For example, in FPC kindreds, smoking increases the relative risk to 19.2 compared to 6.25 in non-smokers [33]. Similarly, individuals with *CDKN2A* mutations experience increased sensitivity to carcinogens with tobacco exposure [4]. These interactions highlight the amplified impact of modifiable risks on an already high genetic predisposition.

### 2.2. Advances in Genetic Testing

#### 2.2.1. Universal Germline Testing

- Recommendations by the National Comprehensive Cancer Network

The NCCN first recommended universal GT for all patients diagnosed with PDAC in 2019 [35]. This groundbreaking recommendation was based on the considerable rate of predisposing PVs in patients with PDAC and the accumulating evidence indicating that typical clinical factors (such as young onset and family history of cancer) are not effective predictors for identifying patients with a PV [36]. For example, a prospective study revealed that 41.8% of patients with PV variants did not meet the classical criteria for GT and were potentially misdiagnosed using these criteria [36].

The NCCN guidelines indicate that germline PVs should be analyzed using a multigene panel that includes *BRCA 1*, *BRCA2*, *CDKN2A*, MMR genes associated with LS (*MSH2*, *MLH1*, *MSH6*, and *EPCAM*), *ATM*, *PALB2*, *STK11*, and *TP53*. *PRSS1* and *SPINK1* are associated with hereditary pancreatitis as well as PDAC and should be included in panels based on clinical features [37].

- Universal Germline Testing: implementation and outcomes of universal testing

Traditionally, clinicians referred selected patients to genetic counseling, where genetic counselors ordered a GT if a hereditary cancer was suspected [38,39]. Based on the recommendations of universal testing, this traditional approach must overcome several challenges, including delays in the first consultation, the increased demand for testing, and the need for a faster turnaround time. These factors are important because these tests have therapeutic implications and due to the poor prognosis of this malignancy [38,40].

Despite recent guideline updates, GT remains significantly underutilized. A study found that only 36% of the patients had a documented discussion about GT, and 78% of those proceeded with the testing [1].

There are different strategies to overcome these challenges. The most commonly used approach for obtaining consent for GT by the treating clinician involves the use of a video educational tool or verbal education. When a PV is identified, the patient or the family is referred to a genetic counselor [40].

The incorporation of videos as an educational tool for patients with PDAC is a key strategy to facilitate pretest education before ordering GT at the point of care. These videos are designed to provide pretest education, covering the role of DNA in genetics, the potential risks and benefits of GT, and its implications for surveillance and treatment. This approach aims to prepare patients before their oncology consultations, enhancing their understanding and engagement in the GT process [41].

The implementation of this video-based education, combined with electronic health record tools and behavioral nudges, led to a high GT acceptance and completion rate, with over 85–100% of eligible patients undergoing testing [41,42,43,44]. This strategy also helped to mitigate disparities in testing uptake among different demographic groups [41,42,44]. Genetic counselors review the results of GT and provide post-test genetic counseling to patients with a significant family history despite negative results, those with PVs, and those with variants of uncertain significance (VUS) who seek clarification [43]. This model avoids overwhelming the genetic counseling services [42].

However, this pathway must overcome challenges to ensure consistency and quality across different cancer types and settings [40,42,44,45,46].

#### 2.2.2. Next-Generation Sequence Panels

NGS technology has revolutionized the genetic evaluation of patients with hereditary PDAC. Historically, GT for these patients followed a stepwise or “cascade” approach, where individual genes were analyzed based on personal and family cancer history. This method, although effective, was time-consuming and limited in scope, as it allowed for the analysis of only one gene at a time [45,46,47]. Studies have demonstrated that multigene panel testing not only saves time but is also more cost-effective compared to the older, gene-by-gene approach [33,47,48,49].

As previously mentioned, the NCCN guidelines currently recommend the use of a multigene panel that includes at least 11 key genes for GT in patients with PDAC [37].

While the standard 11-gene panel has been widely accepted, several studies have investigated the value of expanding the panel. For instance, Gardiner et al. found that using a more comprehensive multigene panel, which included genes like *CHEK2*, *RAD51C*, and *BRIP1*, identified PVs in an additional 11.1% of PDAC patients [50]. Similarly, in a large cohort study by Hu et al., multigene panel testing revealed that 20.73% of patients with PDAC harbored germline PVs in genes such as *ATM*, *BRCA2*, *CHEK2*, *PALB2*, and *CDKN2A*. This study demonstrated the importance of considering a broader gene panel, especially in patients with a family history of cancers such as breast, ovarian, or colorectal cancer, where mutations in these genes are more prevalent [46]. Using broader panels that analyze 133 genes, the rate of PV detection was 33% [47].

However, one major concern with expanded gene panels is the increased detection of VUS [51,52]. Although identified during testing, these variants lack sufficient evidence to determine their impact on cancer risk. As a result, VUS findings can complicate clinical decision-making and increase anxiety for patients, as their implications for cancer susceptibility remain unclear [53].

### 2.3. Early Detection Strategies

#### 2.3.1. Screening for Hereditary PC: Methods and Evidence

Cascade testing of relatives of germline PV carriers identifies individuals at increased risk of developing PDAC, facilitating their enrollment in targeted screening programs and eligibility for risk-reducing procedures, such as salpingo-oophorectomy and mastectomy [54,55].

An HRI is defined as someone with either a lifetime risk of developing PDAC greater than 5% or a fivefold increased relative risk compared to the general population [56,57]. HRIs are classified as those who meet the criteria for FPC or as carriers of a genetic variant linked to a hereditary cancer syndrome, and the two groups have varying PDAC risks. PVs associated with the highest risk of PDAC include STK11/LKB1, CDKN2A, and PRSS1/SPINK1 [57,58]. Additionally, carriers of PVs in BRCA1/2, ATM, PALB2, CDKN2A, MLH1, and APC also present an elevated risk for PDAC, although to varying degrees. Studies emphasize the need for vigilant surveillance in these populations to improve early detection and intervention [59].

The multicenter Cancer of Pancreas Screening-5 (CAPS5) enrolled 1461 HRIs to evaluate the stage at diagnosis and outcome of individuals diagnosed with PDAC and high-grade dysplasia. These individuals underwent annual endoscopic ultrasound (EUS) and/or magnetic resonance imaging (MRI). The detection rate results revealed that one individual was diagnosed with PDAC per year for every 194 screened [60]. Of note, survival outcomes for resectable PDAC were exceptional [60,61,62], with a 3-year survival rate of 85% reported in a screening study involving 366 HRIs [60]. Although the impact of screening on mortality has not yet been studied in clinical trials, these data suggest a potential benefit.

This approach allows the early detection of PDAC, potentially improving prognosis. Given the complexity of PDAC and premalignant lesion management, a multidisciplinary approach is essential, integrating clinical care, genetic counseling, radiology, endoscopy, surgery, and other key specialties to optimize patient outcomes.

The NCCN recommends PC screening for individuals who meet the criteria for FPC or carriers of high-risk germline PVs. The criteria for initiating screening include the following [37]:

- Individuals with two or more first-degree relatives with PC;
- Individuals with a PV in STK11 or CDKN2A, for whom screening should begin at age 35–40 years;
- For carriers of BRCA1/2, ATM, PALB2, MLH1/MSH2/MSH6, EPCAM, or TP3 mutations, screening is not recommended unless they have at least one first- or second-degree relative diagnosed with PDAC. Screening is recommended starting at age 50 or 10 years prior to the youngest age of diagnosis of PDAC in the family.

Currently, no standard screening protocols exist for HRIs, but guidelines recommend an annual EUS and MRI [37,56,57]. A systematic review of 13 studies revealed no serious physical or psychosocial harms associated with PC screening [62]. Despite advances in imaging techniques like MRI or EUS, neither modality is 100% sensitive or specific for PDAC screening [63].

Recently, early screening strategies for HRIs have expanded beyond FPC or carriers of germline mutations. They now also include other high-risk groups, such as individuals with a history of pancreatitis, those with cystic pancreatic lesions (e.g., intraductal papillary mucinous neoplasm or mucinous cystic neoplasm), and adults with new-onset diabetes [64], although this is beyond the scope of this review.

#### 2.3.2. Biomarker Research: Liquid Biopsy

Liquid biopsy, a minimally invasive approach for analyzing circulating tumor biomarkers in body fluids like blood, has emerged as a promising tool for early cancer detection [65,66]. Currently, CA19-9 is a commonly used biomarker for PDAC. However, it is important to note that not all patients show elevated levels of CA19-9, especially those with the Lewis-negative phenotype [67,68]. Additionally, CA19-9 does not have enough sensitivity and specificity to detect early-stage PDAC reliably [65]. Its levels can also be elevated in other conditions, such as pancreatitis and other gastrointestinal malignancies, making its diagnostic accuracy more challenging [68].

Liquid biopsy analyzes circulating tumor cells, circulating tumor DNA (ctDNA), circulating free DNA (cfDNA), miRNAs, proteins, and extracellular vesicles (EVs).

ctDNA is a type of cfDNA released into the bloodstream by tumor cells as a result of apoptosis, necrosis, or active transport by tumor cells [69]. Its main limitation for early detection is its low sensitivity in identifying PDAC during early-stage disease due to the minimal amount of ctDNA present in circulation [69]. Proteins play a key role in tumor progression and have been explored as potential biomarkers for PDAC. However, most single proteins, like TIMP-1, show lower sensitivity and specificity compared to CA19-9 [70].

miRNAs are small non-coding RNAs that are typically 18–25 nucleotides long and play crucial roles in gene regulation at the post-transcriptional level [67,71,72]. Their function is mediated through binding to the 3′ untranslated region of target messenger RNAs, resulting in translational repression or mRNA degradation [71]. They are implicated in different cellular processes, like cancer development and progression [73]. Circulating miRNAs are altered in PDAC patients compared to controls, making them promising diagnostic tools [71,73].

EVs are lipid-bound particles secreted by various cell types, including neurons, epithelial cells, fibroblasts, and cancer cells [74]. EVs encapsulate many molecules, including lipids, nucleic acids (e.g., miRNAs), metabolites, and proteins [65]. EV-derived biomarkers, including RNAs such as miR-21 and proteins like GPC1, demonstrate high diagnostic potential for PDAC, especially in early stages, with sensitivity and specificity reaching 90% (95% CI: 87–93%) and 94% (95% CI: 92–95%), respectively [75].

Panels combining multiple biomarkers outperform single-biomarker assays in sensitivity and specificity, and the different panels have been reviewed elsewhere [65].

One promising approach is the IMMray PanCan-d test, which combines a signature of eight biomarkers with CA19-9 to improve the detection of PDAC in blood samples. In a study involving HRIs, this biomarker panel demonstrated a sensitivity of 85% and a specificity of 98% for detecting PDAC at early stages (I and II) [52]. When patients with normal CA19-9 levels were excluded, the test’s sensitivity and specificity increased to 89% and 99%, respectively, underscoring its potential to outperform CA19-9 alone in early-stage detection [52].

Similarly, the CancerSEEK blood test has emerged as another promising tool for the early detection of various cancers, including PDAC. This test evaluates cfDNA for 16 mutations and combines these findings with the levels of eight circulating proteins. In a cohort of patients with PDAC, CancerSEEK demonstrated a sensitivity of 72% and a specificity of 99% [66]. The sensitivity was lower for patients in stage I and the study lacked the statistical power to provide a detailed sensitivity analysis across different tumor stages [66].

A study led by Nakamura validated a transcriptomic signature based on 13 miRNAs for the detection of PDAC. In the validation cohort, this miRNA signature achieved an AUC of 0.93 for detecting PDAC in early stages (I and II), significantly improving sensitivity and specificity compared to CA19-9 [76]. The use of miRNAs in blood and other bodily fluids represents a non-invasive and highly promising approach for the early detection of PDAC.

The use of liquid biopsy biomarkers for the early detection of PDAC faces several limitations that must be addressed to ensure their clinical applicability. One major challenge is the variability in biomarker expression among patients and tumor subtypes, which affects the consistency and reliability of results across studies [64]. Biomarkers like ctDNA also have a short half-life of 16–114 min, requiring rapid sample processing to prevent degradation and ensure reliable analysis [65,74]. Additionally, clonal hematopoiesis of indeterminate potential or germline mutations in cfDNA might cause false-positive results [65]. Many studies lack validation in independent cohorts, raising concerns about the generalizability of findings [73]. Finally, the high cost and technical complexity of advanced detection techniques and multimarker panels limit their adoption in routine clinical practice [75].

#### 2.3.3. Novel Imaging Techniques: Hyperpolarized 13C Pyruvate-MRS

Studies have evaluated the safety, tolerability, and utility of hyperpolarized 13C pyruvate injection as a diagnostic agent for PDAC and other types of cancers [77,78]. This compound is used in combination with MRS imaging techniques to visualize the metabolism of pyruvate and its metabolites in different tissues, allowing differentiation between areas with normal and abnormal metabolism, particularly in malignant tissues [79,80].

Hyperpolarized 13C-MRS is being investigated for its potential use in diagnosing PDAC and assessing therapeutic responses to chemotherapy. An ongoing phase I study is analyzing the efficacy of hyperpolarized 13C-pyruvate MRS imaging in distinguishing between benign and malignant pancreatic cysts [81]. By analyzing metabolic activity prior to surgical resection, the study aims to correlate these imaging findings with pathological outcomes to improve the detection of invasive carcinoma [81].

### 2.4. Personalized Treatment Strategies

#### 2.4.1. Prevalence of Mutations in Homologous Recombination Genes

Germline mutations in the BRCA1, BRCA2, and PALB2 genes are associated with HRD, which follows DNA damage and increases vulnerability to agents that cause such damage, including platinum-based agents [82]. In the next section, we will discuss studies involving patients with HRD. Therefore, we will first review the prevalence and characteristics of this subgroup of patients.

The prevalence of mutations in HR genes is approximately 15.4% (95% CI, 13–18) as assessed using NGS [83]. However, when evaluated using whole genome sequencing or whole exome sequencing, the prevalence increases to 24–44% [84,85,86]. HR-related alterations are more frequently found in the germline than in somatic cells [87,88,89]. For example, one study reported that 15% of HR mutations were identified through germline testing, compared to 4% using somatic analysis [87].

Core HR genes, including BRCA1/2 and PALB2, are the most frequently mutated (around 15%) and the most extensively studied [88]. Non-core genes also play a role in HR, although they are less studied. In addition, there are ongoing debates regarding the therapeutic implications of many of these mutations [89,90,91]. The prevalence of non-core mutations ranges between 4% and 7%, depending on the specific genes included in the study. Some of these non-core genes include ARID1A, ATM, RAD51, CHEK2, and the Fanconi anemia genes [92,93,94].

Functional inactivation of both copies of an HR gene (biallelic inactivation) may play a crucial role in determining the treatment response [95]. Studies indicate that 89% of germline BRCA mutations in PDAC are associated with biallelic inactivation, compared to approximately 60% for somatic mutations [87]. Biallelic inactivation results in a more profound defect in DNA repair mechanisms, leading to increased sensitivity to platinum-based therapies and poly ADP-ribose polymerase (PARP) inhibitors [92].

#### 2.4.2. Importance of Personalized Approaches

The treatment of patients with susceptibility gene mutations for hereditary pancreatic cancer is primarily based on the use of platinum-based agents, PARP inhibitors, and immune checkpoint inhibitors (ICI). Below, each of these treatments is described.

- Platinum-Based Agents

In breast and ovarian cancers, the sensitivity of patients with germline HRR deficiency to platinum-based agents has been demonstrated [96]. In a retrospective study of 71 patients with PDAC associated with BRCA1/2, it was observed that those with unresectable pancreatic cancer treated with platinum-based agents had significantly longer overall survival (OS) compared to those who received non-platinum agents (22 vs. 9 months, *p* = 0.039) [14]. Additionally, a meta-analysis of six studies comparing platinum-based agents with non-platinum agents in patients with germline BRCA mutations and unresectable pancreatic cancer showed that patients treated with platinum had significantly longer OS (23.7 vs. 12.2 months, mean difference of 10.2 months; 95% CI 5.07–15.37; *p* < 0.001) [97].

Another study of 262 patients who underwent both germline and somatic analyses using the MSK-IMPACT platform revealed that those with HRD experienced significantly better progression-free survival (PFS) following first-line treatment with platinum-based agents compared to those treated with non-platinum agents (12.6 vs. 4.4 months) [87].

- PARP inhibitors

PARP inhibitors have been investigated in cancers with germline BRCA mutations as second-line or later treatments. These agents inhibit PARP’s role in base excision repair, which leads to double-strand breaks in DNA that, due to BRCA1/2 deficiency, cannot be repaired, resulting in cell death [93,94].

In the phase III POLO trial, 154 patients with metastatic PDAC and germline BRCA1/2 mutations who responded to platinum therapy were randomly assigned to receive olaparib or placebo as maintenance therapy. PFS was significantly longer in the olaparib group (7.4 vs. 3.8 months, *p* = 0.004), as was the objective response rate (ORR) (23% vs. 12%) [98]. A phase II study evaluated maintenance rucaparib in patients with PDAC responsive to platinum and a germline or somatic PV in BRCA1, BRCA2, or PALB2. The results were promising, with an ORR of 37% in patients with these mutations [99].

PARP inhibitors were also evaluated as monotherapy. Olaparib and rucaparib were tested in phase II trials involving approximately 20 patients with germline BRCA1/2 mutations and PDAC. The ORRs were 21.1% and 21.7%, respectively [100,101]. Veliparib monotherapy showed disappointing results, with a 0% ORR and median PFS and OS of 1.7 and 3.1 months, respectively. Veliparib’s PARP-trapping activity is lower than that of olaparib and rucaparib [102]. Talazoparib, a next-generation selective PARP inhibitor with the strongest PARP-trapping activity, was also evaluated in a phase I study [103].

PARP inhibitors have also been studied in combination with chemotherapy. Although veliparib has the lowest PARP-trapping activity among PARP inhibitors, it was evaluated in first-line treatment in combination with chemotherapy. In a phase II trial, the addition of veliparib to gemcitabine plus cisplatin did not improve the outcomes. However, the impressive mOS of 15.5–16.4 months supports the use of platinum therapy as a standard approach in this subgroup [104].

Tumor mutational burden and inflammatory activity are associated with DNA damage response (DDR) deficiency. Following the promising results in breast and ovarian cancer, PARP inhibitors are being investigated in combination with ICI for PDAC with germline BRCA1/2 mutations [105]. However, the efficacy of PARP inhibitor treatments in patients with BRCAness (ATM, BAP1, BARD1, BLM, BRIP1, CHEK2, FAM175A, FANCA, FANCC, NBN, PALB2, RAD50, RAD51, RAD51C, and RTEL1), or in HRD PDAC patients, remains unclear [106]. Further studies are needed in this population.

- Immune Checkpoint Inhibitors

In the field of immunotherapy, pembrolizumab has been approved to treat solid tumors with high MSI-H or MMR deficiency [107]. However, response rates to ICI in PDAC are modest and inferior to those observed in other gastrointestinal tumors with MSI-H [108,109].

In a series of 12 patients with refractory PDAC or cholangiocarcinoma with germline HRD treated with a combination of ipilimumab and nivolumab, a 42% ORR was observed, suggesting the potential of HRD as a biomarker of ICI response in PDAC patients [110].

Additionally, the use of niraparib and ipilimumab for maintenance treatment after a response to first-line platinum therapy has demonstrated potential efficacy. Among the seven patients with BRCA or PALB2 variants, the mPFS was 10.4 months (95% CI, 1.5–19.2), and the mOS was 38 months (95% CI not estimable) [111].

## 3. Discussion

The benefits of universal GT in PDAC have been previously demonstrated. When implementing point-of-care GT, various challenges emerge. One of these challenges is ensuring that clinicians are adequately trained to perform high-quality pre-test counseling. Adequate training ensures that patients are fully informed about the implications of GT, potential outcomes, and the process itself. Additionally, the interpretation and management of VUS represent another major challenge. It is crucial that GT results are reviewed by genetic services to provide accurate interpretations and follow-up recommendations.

Furthermore, although oncologists are capable of delivering negative results, thereby alleviating the burden on genetic counseling services, there remains debate about who should provide post-test counseling for VUS. Some argue that clinicians can handle these consultations, while others believe that geneticists should manage these discussions due to their complexity. Continued research into VUS and their classification is essential, as this will advance our understanding and ultimately improve patient care.

The question of which genes should be included in GT remains a controversial topic. It is well established that increasing the number of genes analyzed enhances the detection rate. However, there is ongoing uncertainty regarding the therapeutic implications of these alterations and the risk of PDAC development in families carrying low-to-moderate penetrance genes. Further research is needed to elucidate how these findings should influence clinical decision-making.

Additionally, somatic mutation testing in patients with PDAC is also recommended based on current guidelines. Contrary to what might be assumed, both germline and somatic testing have proven to be complementary, providing essential and often distinct information critical for the appropriate management of these patients [112].

Although PDAC screening has shown potential in improving early detection rates and outcomes, several controversies remain. One of the major controversies is determining who should undergo screening. Current evidence suggests that individuals with familial aggregation, even in the absence of a known pathogenic mutation, should be included in screening programs. Carriers of *CDKN2A* mutations often present with more aggressive tumors, and their resectability rates remain lower despite undergoing annual screening. This has raised the question of whether the frequency of screening should be increased to biannual intervals for this subgroup [113,114,115]. Additionally, there is ongoing debate about whether individuals with moderate risk for PDAC, such as *BRCA2* or *ATM* mutation carriers without a family history of the disease, should be considered candidates for screening.

The management of indeterminate findings, such as subcentimeter lesions, pancreatic duct dilatation, or parenchymal atrophy, remains unclear. These findings can lead to unnecessary surgical resections, adding complexity to the decision-making process. Therefore, screening programs should ideally be conducted within the framework of clinical trials with multidisciplinary collaboration or in specialized centers.

The future of PDAC screening lies in refining risk stratification models by integrating genetic, clinical, and environmental factors to better identify HRIs. Advances in biomarkers, such as ctDNA, miRNA, or EVs, along with improvements in imaging technologies, hold promise for earlier detection. Personalized surveillance protocols based on individual risk factors and biomarker profiles may improve screening accuracy, balancing early detection with the risk of overdiagnosis, ultimately leading to more effective and targeted screening strategies.

Patients with HRD who receive platinum-based chemotherapy exhibit significantly improved outcomes. However, up to 25% of patients with HRD do not receive such treatment [90]. This is likely due to the deterioration of the patient’s condition by the time HRD status is identified [90]. This underscores the critical need for early germline and somatic testing, ensuring that HRD-positive patients are identified and treated before clinical progression limits therapeutic options. Interestingly, clinical benefit appears to be comparable in patients with either germline or somatic mutations [82].

There are numerous genes involved in the DDR pathway, and several studies suggest that platinum therapy may benefit patients with mutations in genes such as *ATM* and *ATR*, those within the MRN complex (e.g., *RAD50*), and Fanconi anemia core genes. However, further investigation is required. Additionally, evidence suggests that patients with biallelic loss exhibit greater genomic instability and are more likely to benefit from platinum-based therapy compared to those with monoallelic loss [91]. This genomic instability may also influence responses to PARP inhibitors and immunotherapy. Efforts to standardize the definition of HRD in PDAC include studying the utility of genomic scarring as a surrogate marker, which could be employed in clinical trials to better stratify patients.

Maintenance therapy with olaparib in patients with germline *BRCA1/2* mutations has demonstrated improved PFS in platinum-sensitive patients [98]. However, emerging strategies aim to enhance these outcomes. For instance, the phase II POLAR trial reported a promising 35% ORR for the combination of olaparib with pembrolizumab in patients with either germline or somatic mutations in *BRCA1/2* and *PALB2* [116]. Although the results of immunotherapy in PDAC have generally been disappointing, recent data on the use of ipilimumab–nivolumab in HRD mutant PDAC and the combination of niraparib with ipilimumab in platinum-responsive patients show promise for identifying subgroups that may benefit from these treatments. Lastly, there is growing interest in novel therapeutic strategies targeting DDR-related pathways, such as ATM, ATR, and WEE1 inhibitors, which could further expand treatment options for HRD-positive patients.

## 4. Conclusions

The implementation of GT in PDAC is of vital importance. However, the timely and effective application of this approach requires the modification of genetic counseling services. PDAC screening has shown promising results, particularly regarding its potential for early diagnosis. Nevertheless, further research is needed to improve early detection methods. The treatment of patients with HRD mutations opens a window of opportunity to improve the prognosis of PDAC patients.
